# Flare Removal Model Based on Sparse-UFormer Networks

**DOI:** 10.3390/e26080627

**Published:** 2024-07-25

**Authors:** Siqi Wu, Fei Liu, Yu Bai, Houzeng Han, Jian Wang, Ning Zhang

**Affiliations:** 1 School of Science, Beijing University of Civil Engineering and Architecture, Beijing 100044, China; 2102520021006@stu.bucea.edu.cn; 2 School of Geomatics and Urban Spatial Informatics, Beijing University of Civil Engineering and Architecture, Beijing 100044, China; hanhouzeng@bucea.edu.cn (H.H.); wangjian@bucea.edu.cn (J.W.); zn20040924@163.com (N.Z.)

**Keywords:** image flare removal, sparse-UFormer, multi-scale information, top-k sparse attention, structural similarity index

## Abstract

When a camera lens is directly faced with a strong light source, image flare commonly occurs, significantly reducing the clarity and texture of the photo and interfering with image processing tasks that rely on visual sensors, such as image segmentation and feature extraction. A novel flare removal network, the Sparse-UFormer neural network, has been developed. The network integrates two core components onto the UFormer architecture: the mixed-scale feed-forward network (MSFN) and top-k sparse attention (TKSA), creating the sparse-transformer module. The MSFN module captures rich multi-scale information, enabling the more effective addressing of flare interference in images. The TKSA module, designed with a sparsity strategy, focuses on key features within the image, thereby significantly enhancing the precision and efficiency of flare removal. Furthermore, in the design of the loss function, besides the conventional flare, background, and reconstruction losses, a structural similarity index loss has been incorporated to ensure the preservation of image details and structure while removing the flare. Ensuring the minimal loss of image information is a fundamental premise for effective image restoration. The proposed method has been demonstrated to achieve state-of-the-art performance on the Flare7K++ test dataset and in challenging real-world scenarios, proving its effectiveness in removing flare artefacts from images.

## 1. Introduction

Ideally, a flare-free image is produced by light reflected from objects in the scene or directly from a light source, effectively collected by the camera lens and fully focused on the image sensor [1]. However, in outdoor scenes, camera lenses are often contaminated by fingerprints, dust, etc., and a small amount of incident light scatters and reflects in an unintended direction within the lens, creating an unwanted flare effect.

**Figure 1:**
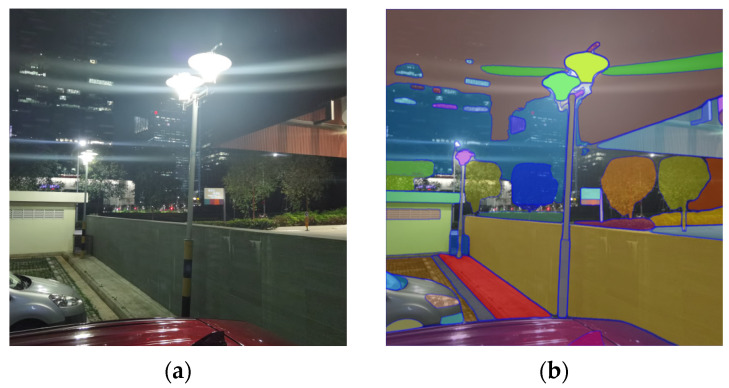
(**a**) An image contaminated by a flare near a street light. (**b**) The result of the visual sensor’s semantic understanding based on Picture (**a**). It is evident that the flare has been inaccurately segmented into the actual scene.

**Figure 2:**
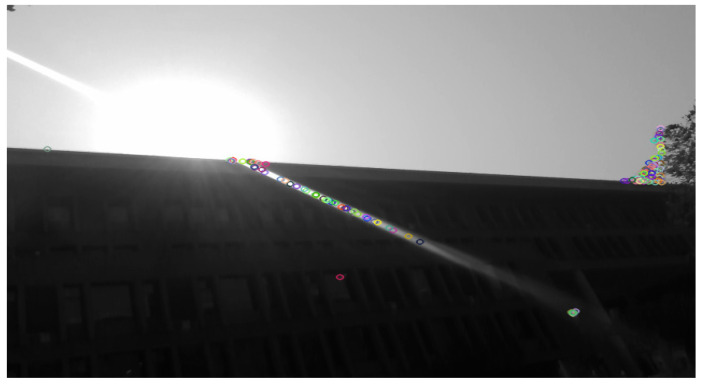
This figure is based on the ORB-SLAM2 system running in real-time for feature extraction. Due to the interference from the flare, the feature points are incorrectly extracted along the flare streaks, while the house with significant texture changes has almost no feature points extracted.

Flares can be divided into two types based on the distribution of stray and reflected light on the sensor and its effect on the image: stray flare and reflected flare [2]. Stray flare usually appears as bright, shiny streaks in the image, which can obscure the details of the original scene. Reflected flare, on the other hand, creates polygonal or star-shaped maps in the image, and the former is more likely to affect tasks that rely on visual sensors, such as semantic segmentation and image feature extraction. Specifically, as depicted in Figure 1, the presence of flare interferes with the computer’s semantic understanding. Figure 2 illustrates that, if an image contains flare, it is highly likely to disrupt the extraction of feature points, potentially leading to the failure of downstream tasks.

In the context of information theory, flare represents unwanted noise that reduces the effective entropy of the image. Entropy, a measure of information content, is crucial for tasks such as semantic segmentation and feature extraction, where the clarity and detail of the image is paramount. The presence of flare reduces entropy by introducing artefacts that obscure the true information contained in the image. Therefore, removing the flare not only improves visual quality, but also restores the information content and entropy of the image, ensuring the more accurate and reliable performance in downstream tasks.

Existing methods to combat or reduce the effects of flare fall into three broad categories. The first is hardware-based flare reduction methods. Physical components and mechanisms within the camera and lens system are used to reduce or eliminate the adverse effects of lens flare. One method is the use of anti-reflective coatings [3], also known as AR coatings, which are often applied to the surface of optical components such as lenses or camera filters to reduce reflections and flare from bright light. Such coatings reduce the overall reflection by creating phase differences between multiple reflected light waves, using the principle of phase cancellation interference [4]. The results of this enhancement have been good in terms of improving the image contrast and reducing the flare and reflections. However, anti-reflective coatings can only reduce the unwanted effects of light of certain wavelengths and angles of incidence, and are costly. Another approach is to use lens hoods, which are an important accessory for cameras and lenses to improve the quality of photographs. Typically mounted on the front of the camera lens, lens hoods essentially block and shield the lens from unwanted light sources by creating a physical barrier in front of the lens to prevent interference from extremely angled or non-axial light. While hoods must be designed to match the field of view and focal length of the lens and optimised in shape and length to effectively block unwanted light, they can degrade the image quality in low light or controlled lighting conditions, and significantly increase the cost of ownership, in addition to the fact that these solutions can increase the weight of the optical system, are difficult to manufacture, and are not flexible enough to adapt to different environments and photographic scenarios once integrated.

The second is based on conventional image processing to reduce flare [5]. Seibert et al. [6] suggested that occlusion flare can be eliminated by knowing the point spread function (PSF) of the image intensifier and applying mathematical inverse convolution. The parameters of the PSF are denoted by ρ and k, where ρ is the fraction of intensely scattered light in the image intensifier and k is the measure of the average path of the scattered photons. These two parameters can be determined by a least-squares fit of the contrast ratio and guide disc diameter. However, this method is based on the assumptions of circular symmetry and spatial invariance of the image intensifier PSF and may not be applicable to all cases of flare. Floris Chabert et al. [7] proposed an automated method for detecting lens flare, in particular reflective flare, based on a single input image. The method includes a custom flare detection algorithm based on the OpenCV concept [8] and uses a hybrid patching method called exemplar-based inpainting [9]. The detection algorithm is divided into five steps: multiple thresholding, contour detection, blob merging, blur candidate filtering, and blur mask calculation. The recovery phase uses exemplar-based patching by selecting windows around the flare and then running the algorithm until all pixels are repaired. However, this approach is limited to certain types of flare, is difficult to generalise to other types of flare, and may incorrectly mark any saturated blob as a flare.

Third, image flare removal based on the deep learning approach: the development of computer vision technology promotes the progress of image processing technology, Zhang et al. [10,11,12] took semi-supervised and other ways to remove image reflections, Qian R. et al. [13,14] used GAN network to de-rain the image, but the progress of image flare removal algorithms is slow, and the main reason for this is that it is more difficult to collect the dataset of pairs of flaring images; until 2021, Wu et al. [1] used a physics-based approach to synthesise the first flare dataset in the laboratory and used U-network training to achieve flare removal from a single image, but there is a large gap between the synthesised flare in this dataset and the flare in the real scene, and the U-network removal is ineffective. To solve the problem of limited data, Qiao et al. [15] trained the GAN network to remove the flare based on an encoder–decoder architecture and introduced a circular consistency constrained loss function for the unpaired data generated by the network, but the training of the network was extremely unstable and the flare removal effect was poor. Dai et al. [2] created a new nighttime flare dataset, Flare7K, which is derived from real-world nighttime lens flares and uses different types of lenses and different light sources as reference images, but since the synthetic dataset does not include the complex degradation caused by diffraction and dispersion in the lens system, Dai et al. introduced another real-world flare dataset, Flare-R [16], which consists of 962 real-world flare patterns and replicates common the lens contaminants encountered in daily use to make the Flare7K dataset diverse, making the neural network particularly effective at removing complex degradation around light sources. Based on this dataset, Dai et al. constructed several baseline models, including HINet [17], MPRNet [18], Restormer [19], and Uformer [20]. The results show that the Uformer network is the most effective at removing flare. Subsequently, Yousef Kotp et al. further used the Uformer network and combined it with the depth information of the image to achieve better results.

In 2023, Xiang Chen et al. [21] designed the sparse transformer network for single image rain removal with good results, and inspired by the mixed-scale feed-forward network (MSFN) module in this model and top-k sparse attention (TKSA), we design the sparse transformer network for single image flare removal and add the SSIM structural loss function for model training. The experimental results show that our method outperforms existing techniques, and our contributions can be summarised as follows:

1. In order to develop a novel flare removal method, we replaced the W-MSA and LeFF modules in the traditional UFormer encoding structure with the TKSA and MSFN modules.
2. We design a novel loss function and achieve a significant improvement in the experimental quantitative metrics.
3. We perform extensive experiments on different benchmarks to compare our method with state-of-the-art methods both qualitatively and quantitatively.

## 2. Model

This section describes the main architecture of the Sparse-Uformer Flare Removal Network, including the modular details of the TKSA and MSFN.

### 2.1. Overall Pipeline

**Figure 3:**
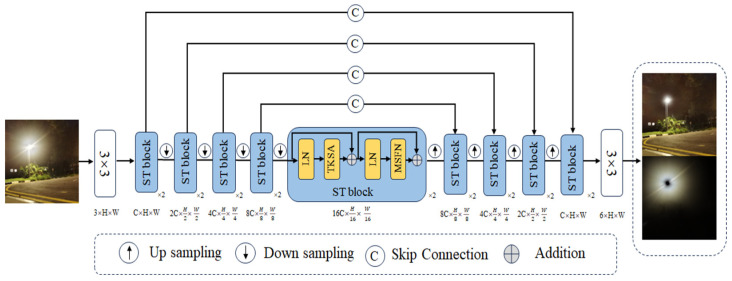
The overall framework of our model: First, there is the encoding phase, where we use 4 encoders to extract image features; each encoder contains 2 sparse transform modules with a downsampling layer, which first reconstructs the 1D feature map output from the transform into a 2D form, and then downsamples it by a 4 × 4 convolution with a step size of 2, while doubling the number of channels. Next, comes the bottleneck phase, which again uses 2 sparse transform modules to integrate all the high-level features from the coding phase and capture global dependencies. The decoder also contains 4 stages, each consisting of an upsampling layer and 2 sparse transformer modules, similar to an encoder. The feature map is then upsampled using a 2 × 2 inverse convolution with a step size of 2 to increase the feature resolution and reduce the number of channels, and finally the upsampled features are combined with the features from the corresponding coding stage to reconstruct the image.

We make improvements to the UFormer network and design the Sparse UFormer neural network model, in which the Sparse Transformer module effectively removes the flare from the image more. The basic framework of the network extends the U-shaped architecture of the UFormer network and the jump connection design of the encoding and decoding layers. Figure 3 shows the overall framework of the Sparse UFormer network.

In the Sparse UFormer network, the coding layer, the decoding layer, and the bottleneck layer are all constructed on the basis of the sparse transformer module. Unlike the Lewin module of UFormer, the Sparse Transformer blocks adopts a sparse attention mechanism to focus on the most informative part of the image, and this design not only improves the model’s ability to detect and remove flare, but also reduces the interference of irrelevant information on the network’s performance. The redesigned feed-forward network incorporates multi-scale features, which effectively improves the network’s ability to handle flare features at different scales. In addition, the algorithm modifies the network output dimension so that the network can directly predict a six-channel output, including a three-channel RGB image after flare removal and a three-channel RGB flare image, ensuring that these two images are summed to reconstruct the original input image and using the reconstruction loss to monitor the quality of the final output. Specifically, given a flare-contaminated image $I_{input}\in R^{3\times H\times W}$, the network first extracts the underlying feature $X_{0}\in R^{C\times H\times W}$ by a 3 × 3 convolutional layer supported by a LeakyReLU activation function:

(1) $$X_{0}=LeakyReLU\left({Conv}_{3\times 3}\left(I_{input}\right)\right).$$

where C is the number of image feature maps and H and W are the image dimensions. Second, according to the U-shaped architecture, the feature $X_{0}$ is processed by four encoders, each encoder contains two sparse transformer blocks with a downsampling layer, the downsampling layer first reconstructs the one-dimensional feature $X_{0}$ output from the transformer into a two-dimensional form, and then downsamples it by a 4 × 4 convolution with a step size of 2, while doubling the number of channels. The feature map $X_{k,encoder}\in R^{{(2}^{k}C)\times \frac{H}{2^{k}}\times \frac{W}{2^{k}}}$ of the encoder output at the kth stage:

(2) $$X_{k,encode}=Downsample\left(SparseTransformers\left(X_{k-1,encode}\right)\right).$$

In the bottleneck stage, two sparse transformer blocks are used again to integrate all high-level features from the encoding stage, capturing global dependencies:

(3) $$X_{bottleneck}=SparseTransformers\left(X_{4,encode}\right).$$

where the feature map $X_{bottleneck}\in R^{{(2}^{4}C)\times \frac{H}{2^{4}}\times \frac{W}{2^{4}}}$, in terms of feature reconstruction, the decoder also contains four stages, each consisting of an upsampling layer and two sparse transformer blocks. The feature map is then up-sampled by a 2 × 2 inverse convolution with a step size of 2 to improve the feature resolution and reduce the number of channels, and finally the up-sampled features are fed into the next stage along with the features from the corresponding encoding stage. The decoder at stage k outputs a feature map $X_{k,decoder}\in R^{{(2}^{\left(4-k\right)}C)\times \frac{H}{2^{\left(4-k\right)}}\times \frac{W}{2^{\left(4-k\right)}}}$:

(4) $$X_{k,decoder}=SparseTransformers\left(Upsample\left(X_{k-1,encode}\right)⊕X_{4-k,decode}\right).$$

where ⊕ denotes the feature fusion operation, the features are reshaped into a 2D form, and finally, the reconstructed image and the flare image $I_{recon}\in R^{6\times H\times W}$ are obtained by 3 × 3 convolutional layers.

### 2.2. Sparse Transformer Block

**Figure 4:**
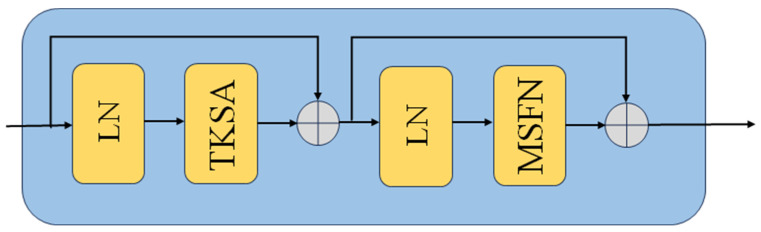
The sparse transformer block. The encoding structure is consistent with that of the stand Uformer model. The difference is that we replaced the traditional non-overlapping window multi-head self-attention (W-MSA) and the locally enhanced feed-forward network (LeFF) with TKSA and the MSFN, respectively.

**Figure 5:**
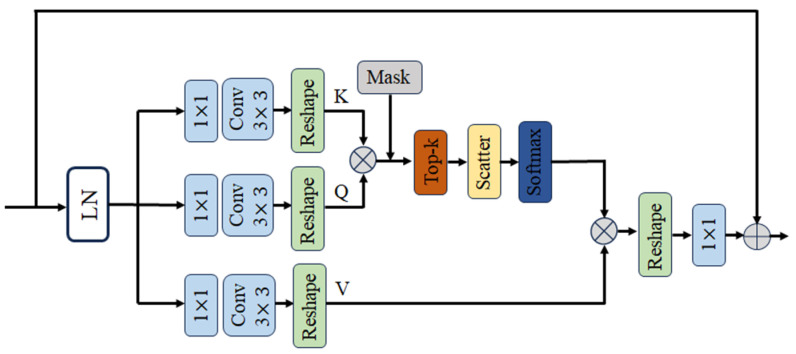
The architecture of the top-k sparse attention module. The module is divided into five main steps: (1) image channel coding; (2) cross-channel self-correction; (3) calculation of pixel pair similarity; (4) top-k score selection; and (5) TKSA output features.

**Figure 6:**
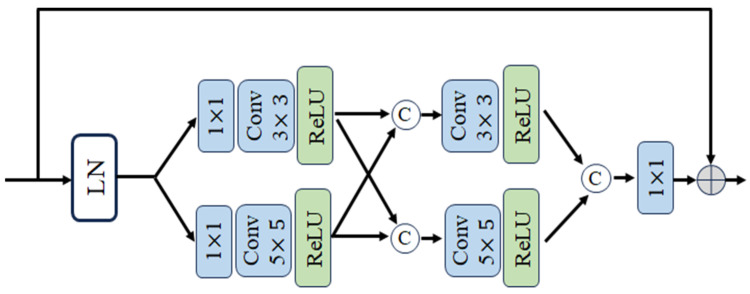
The architecture of the top-k sparse attention module. The module is divided into four main steps: (1) feature expansion; (2) multiscale deep convolution; (3) nonlinear activation and feature fusion; and (4) residual linking and output remodelling.

We adopt the Sparse Transformer block designed by Xiang Chen et al. [21]. In it, we retain the two core components of the Top-k sparse attention module and the multi-scale feedforward convolutional network. Figure 4, Figure 5 and Figure 6 show the sparse transformer block, the TKSA module, and the MSFN module, respectively. Given the output feature $X_{k-1}$ of the (k − 1)st module, the model first evaluates the amount of information in each image block using the sparse attention mechanism, and then selects the most meaningful image blocks to compute the attention scores, thus highlighting these focal regions in the feature representation. A multiscale convolution strategy is then used to enhance the network’s ability to process the salient features of different region sizes in the image, allowing the network to capture multi-scale image features in the feed-forward process:

(5) $$X_{k}^{\prime }=TKSA\left(LN\left(X_{k-1}\right)\right)+X_{k-1},$$

(6) $$X_{k}=MSFN\left(LN\left(X_{k}^{\prime }\right)\right)+X_{k}^{\prime }.$$

#### 2.2.1. Top-k Sparse Attention Module

In contrast to the conventional multi-head attention module, the TKSA module arranges the scores of each row of the matrix M after the attention matrix M $\in R^{C\times C}$. Subsequently, the module retains the highest scores in each row, with the proportion of which being determined by the tuneable parameter k. This parameter enables the model to dynamically control the degree of sparsity, thereby allowing it to flexibly adjust the focus of the attention in this interval according to the different image characteristics and flare conditions. This approach ensures that the model’s attention is focused on those feature pairs that are most relevant, which not only reduces the computational burden of the model, but also allows for a more focused analysis of the most important features. The expression is as follows:

(7) $${{[M}_{top-k}]}_{ij}=\left\{\begin{array}{c} M_{ij},M_{ij}<t_{i}\\ \;0\;,Else \end{array}\right..$$

where $M_{ij}$ is the value of matrix *M* in row *i*, column *j*, and $t_{i}$ is the minimum value of the attention matrix *M* in row *i* that is greater than the *k*th quantile.

The sparse attention weight matrix $M_{top-k}$, selected by Top-k, is then softmax normalised and multiplied with the value *V* to obtain the sparse attention output. This is expressed as follows:

(8) $${Attention}_{n}(X^{i})=\mathrm{softmax}\left(\frac{M_{top-k}}{\sqrt{d_{n}}}\right)\cdot V_{n}.$$

where $d_{n}$ is the feature dimension size of each attention head. Finally, we concatenate all the outputs of the multi-head attention and obtain the final output features through a linear projection layer.

#### 2.2.2. Multi-Scale Feedforward Convolutional Network Module

Existing studies [18,22] typically use single-scale deep convolution in feed-forward networks to improve the local feature extraction, but these methods tend to ignore the correlation between features at different scales. Multi-scale feature representation has been shown to be very effective in removing complex image clutter, such as removing raindrops [23], so based on this concept, this paper incorporates a dual-path multi-scale deep convolution MSFN network to enhance the network’s ability to capture glare features at different scales.

The MSFN module employs two parallel deep convolutional paths, each utilising a distinct convolutional kernel size. The first path employs a 3 × 3 convolutional kernel, while the second employs a 5 × 5 convolutional kernel.

(9) $$X_{k}^{a1}=Dw{Conv}_{3\times 3}\left(X_{k}\right),$$

(10) $$X_{k}^{b1}=Dw{Conv}_{5\times 5}\left(X_{k}\right).$$

where $Dw{Conv}_{k\times k}$ denotes the depth convolution with convolution kernel size *k*. This configuration enables the module to capture local features at varying scales. Subsequently, the outputs of the two parallel paths are activated by the ReLU activation function, facilitating the integration of feature information at different scales. Subsequently, the respective outputs of the feature maps are spliced together in the channel dimension in order to integrate the feature information at different scales. These parts are then subjected to a second deep convolution process, after which the channels of the feature maps are merged again, thus effectively integrating feature information at different scales. The expression for this is as follows:

(11) $$X_{k}^{a2}=Relu(Dw{Conv}_{3\times 3}\left(Cat\left[Relu\left(X_{k}^{a1}\right),Relu\left(X_{k}^{b1}\right)\right]\right)),$$

(12) $$X_{k}^{b2}=Relu(Dw{Conv}_{5\times 5}\left(Cat\left[Relu\left(X_{k}^{a1}\right),Relu\left(X_{k}^{b1}\right)\right]\right)).$$

where Cat[∙] denotes channel connection.

## 3. Loss Function

We follow Dai et al.s’ approach [16], where we consider the SSIM structural loss function in addition to the flare loss function, the background loss function, and the reconstruction loss function. The total loss is defined as:

(13) $$L_{total}=\alpha_{1}L_{F}+\alpha_{2}L_{base}+\alpha_{3}L_{rec}+\alpha_{4}L_{SSIM}.$$

The flare loss function is defined as:

(14) $$L_{F}={\left\|{\dot{F}}_{0}-F_{0}\right\|}_{1}+\sum_{l}\lambda_{l}{\left\|\Phi_{l}\left({\dot{F}}_{0}\right)-\Phi_{l}\left(F_{0}\right)\right\|}_{1}.$$

The background loss function is defined as:

(15) $$L_{base}={\left\|{\dot{I}}_{0}-I_{0}\right\|}_{1}+\sum_{l}\lambda_{l}{\left\|\Phi_{l}\left({\dot{I}}_{0}\right)-\Phi_{l}\left(I_{0}\right)\right\|}_{1}.$$

The reconstruction loss loss function is defined as:

(16) $$L_{rec}=\left|Clip(I_{0}⊕F_{0})-Clip({\dot{I}}_{0}⊕{\dot{F}}_{0})\right|.$$

The SSIM structural loss loss function is defined as:

(17) $$L_{SSIM}=1-SSIM\left(x,y\right),$$

(18) $$SIM\left(x,y\right)=\frac{\left(\mu_{x}\mu_{y}+c_{1}\right)\left(2\sigma_{xy}+c_{2}\right)}{\left(\mu_{x}^{2}+\mu_{y}^{2}+c_{1}\right)\left(\sigma_{x}^{2}+\sigma_{y}^{2}+c_{2}\right)}.$$

where $\alpha_{i}$ is a weight coefficient; $F_{0}$ and ${\dot{F}}_{0}$ denote the flare image in the training set and the flare image predicted by the network, respectively; $I_{0}$ and ${\dot{I}}_{0}$ denote the background image in the training set that is not contaminated by flare and the background image predicted by the network, respectively; and $\Phi_{l}(\cdot )$ denotes the output of the pre-trained model at the layer specified by the feature map. ⊕ denotes the addition operation in the linearised gamma decoding domain; and Clip(⋅) is a cropping function that ensures that the pixel values of the images are within a reasonable range. *x* and *y* denote the original and flare-removed images; $\mu_{x}$ and $\mu_{y}$ are the pixel averages of the respective images; $\sigma_{x}^{2}$ and $\sigma_{y}^{2}$ are the image variance; $\sigma_{xy}$ is the covariance of the two images; and $c_{1}$ and $c_{2}$ are small constants that are added in order to avoid a denominator being zero and small constants added.

## 4. Dataset

We trained the Sparse-UFormer network glare removal model by selecting the 24K Flickr dataset [12] of background images without glare contamination, which contains 23,949 images, and the Flare7K++ dataset [16] of glare images, which contains 7000 virtual glare images and 962 real-world glare images and their corresponding light source images in the real world. First, the virtual and real glare images and their corresponding light source images are sampled from the glare dataset with a 50% probability, and then inverse gamma corrected on them and the background images to recover the linear luminance level. We then apply a series of on-the-fly transformations to the glare data images, including rotation, translation, cropping, scaling, blurring, and mirroring. Next, we apply random global colour shifts to the images with added glare to simulate the glare illuminating the entire scene, and for the background images, we randomly adjust their RGB values and add Gaussian noise to them to increase the realism and diversity of the images. Finally, the paired images after these preprocessings are input to the neural network for training.

## 5. Experiment and Result

### 5.1. Parameter Settings

The experiments were conducted on an NVIDIA TESLA V100 32G graphics card (Santa Clara, CA, USA), and the PyTorch framework was employed to train the model. Furthermore, the optimiser was selected as Adam, the learning rate was set to 0.0001, and two momentum parameters, $\beta_{1}$ = 0.99 and $\beta_{2}$ = 0.99, were set for smoothing the gradient update to improve the training stability, and the learning rate scheduler was adopted as MultiStepLR; after every 200,000 iterations, the learning rate will be multiplied by 0.5 for attenuation, and the total number of iterations is set to 800,000. In addition, the loss function weights $\alpha_{i}$ are set to 0.5, 0.5, 1, 0.5, respectively. The transformation parameters for training data enhancement are presented in Table 1.

### 5.2. Evaluation Metrics

**Figure 7:**
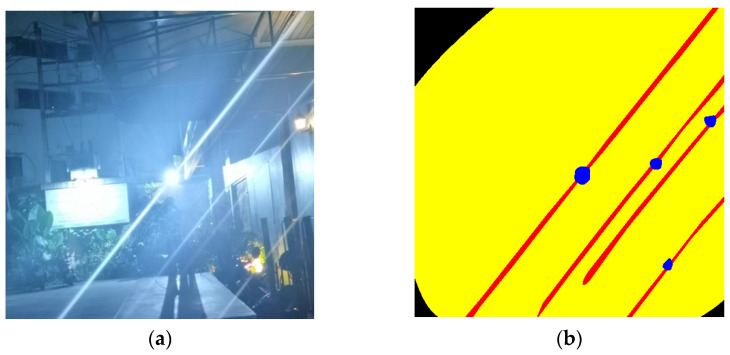
Masking diagram for G-PSNR and S-PSNR evaluation. Figure (**a**) shows an image contaminated by a flare. Image (**b**) is the corresponding mask map. The yellow part is the halo mask, the red part is the bright streak mask, and the blue part is the light source mask.

In the field of image denoising, the main image quality evaluation indexes after denoising are peak signal-to-noise ratio (PSNR), structural similarity index measure (SSIM), learned perceptual image quality loss (LPIPS), and perceptual image patch similarity (LPIPS). For the image glare removal task, Dai et al. [16] proposed glare peak signal-to-noise ratio (G-PSNR) and streak peak signal-to-noise ratio (S-PSNR) for glare on the basis of PSNR. Figure 7 shows the mask map they manually drew to evaluate the flare region of the test image. G-PSNR calculates the PSNR value of the original image and the image after the removal of the flare in the flare region (including the halo and the bright streak), and S-PSNR calculates the PSNR value of the original image and the image after the removal of the flare in the bright streak region, and we selected the above total of five metrics to quantitatively evaluate the quality of the flare-removed image.

### 5.3. Experimental Result

#### 5.3.1. Quantitative Assessment Results

To verify the effectiveness of our proposed model. We evaluated 100 real flare images in the Flare7K++ test dataset, and selected the more typical methods in the field of image denoising to compare with the results of this paper, and they all used the same dataset for their training. We used the five metrics introduced in Section 5.2 (PSNR, SSIM, G-PSNR, S-PSNR, and LPIPS) to evaluate the model performance, where the higher PSNR, SSIM, G-PSNR, and S-PSNR and the lower LPIPS indicate the better flare removal effect. Table 2 shows the quantitative evaluation results of our models on the real world, which show that our designed Sparse Uformer model outperforms U-Net [24], HINet [17], MPRNet [18], Restormer [19], the baseline model UFormer [20], the UFormer with normalised depth [21] in all metrics. The PSNR score is 0.314 higher than the second place, the SSIM score is 0.009 higher than the second place, the LPIPS score is 0.009 lower than the second place, the G-PSNR score is 0.256 higher than the second place, and the S-PSNR score is 0.682 higher than the second place, which shows that our model removes the bright streak flare more effectively.

We also evaluated 100 synthetic flare images in the Flare7K++ test dataset. Most models were not tested on synthetic data due to the inclusion of real flares in the training set, and we selected the open source UFormer [20] baseline model as well as the UFormer with normalised depth [21] model for testing. Table 3 shows the test results on the synthetic dataset, and the results show that our model achieves the best results in the PSNR, SSIM, LPIPS, and S-PSNR metrics.

#### 5.3.2. Qualitative Assessment Results

**Figure 8:**
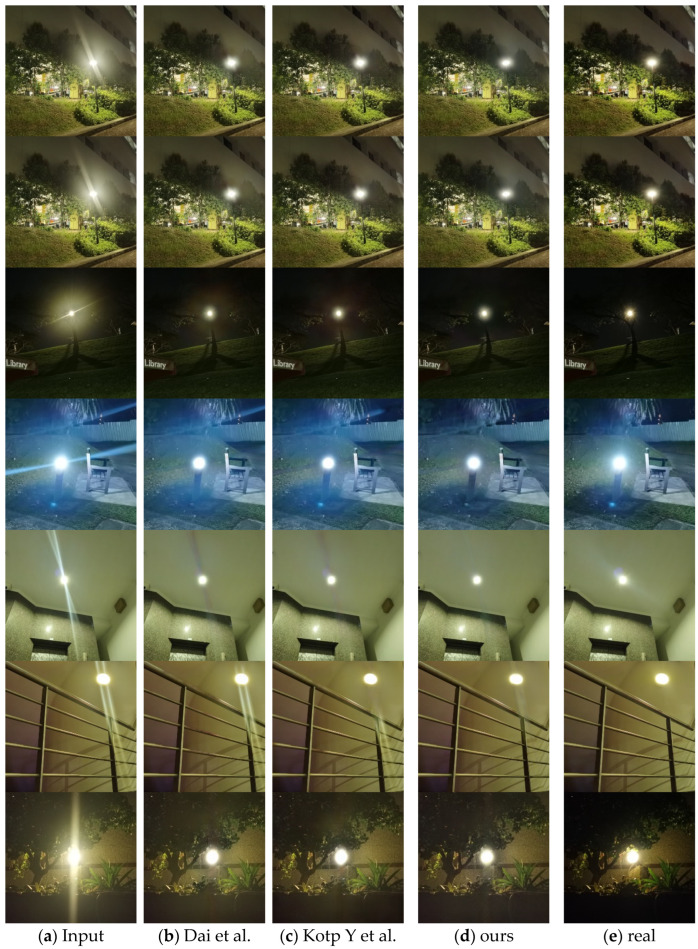
Comparison of the test results of different methods on Flare7k++ real dataset. Picture (**a**) shows the input image containing flares; Picture (**b**) shows the experimental results of Dai et al. [20] using the UFormer model; Picture (**c**) shows the experimental results of Kotp Y. et al. [25] using the UFormer model and incorporating the depth information; Picture (**d**) shows the experimental results using our Sparse UFormer model; Picture (**e**) shows the results of the real no-flare experiment.

The improvement of visual perception by flare removal is also obvious, and we selected seven representative flare images of real scenes in the Flare7k++ dataset, and also used the current state-of-the-art flare removal UFormer baseline model and the UFormer with normalised depth model to compare with ours, and the results of the experiment are shown in Figure 8, where the first column is the real flare image, the penultimate column is the image after flare removal with our Sparse UFormer model, and the last column is the real flare-free image. It can be seen that the flare removal effect of our model is more obvious and closer to the real scene.

**Figure 9:**
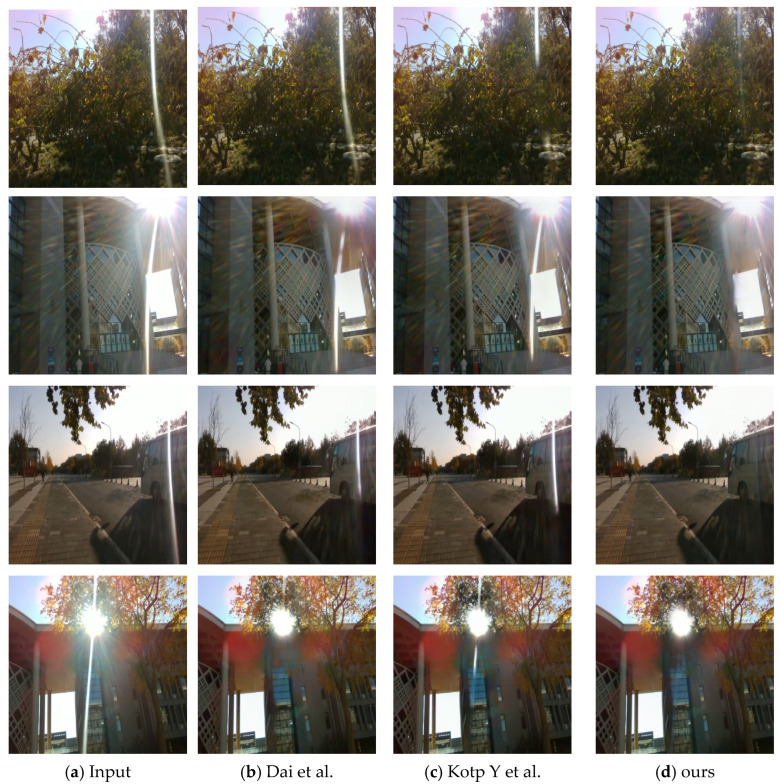
Comparison of the results of different methods for strong daytime flares. Picture (**a**) shows the input image with flares; Picture (**b**) shows the experimental results using the UFormer model by Dai et al. [20] Picture (**c**) shows the experimental results using the UFormer model combined with depth information by Kotp Y et al. [25] Picture (**d**) shows the experimental results using our Sparse UFormer model.

In order to further verify the capability of our model, we also took some images with a high flare challenge at noon for testing, and each image has very bright streaky flare. We also selected the current state-of-the-art flare removal model to do the comparison experiment. Figure 9 shows our results, where the first column exhibit is the input image, the last column is our algorithm flare. After the processing of our Sparse-UFormer algorithm, the degree of flare stripe removal is more obvious, and the visual effect is more excellent, which further proves the applicability of the proposed flare removal model in practical application scenarios.

#### 5.3.3. Ablation Study

To verify the validity of the Sparse-transformer module and the SSIM structure loss, we set up an ablation experiment to remove the Sparse-transformer module and the SSIM structure loss, respectively. The model with the Sparse-transformer module removed is still modelled with the LeWin transformer module. The experiment investigates the two components on the image artifacts removal performance. The experimental results are presented in Table 4. The first row uses UFormer as the baseline model, which has the worst results for each evaluation. The second row removes the SSIM structural loss and retains the Sparse-transformer module based on our modelling. Compared to the first row, the model results have improved. This demonstrates the effectiveness of the sparse transformer module. In the third row, the SSIM structural loss was retained and the Sparse-transformer module was removed. Here, the SSIM score has improved compared to the first and second rows, which proves the effectiveness of the SSIM structure loss. In the fourth row, we use the full Sparse-transformer model and the model’s scores on all five metrics are improved. This fully confirms that the two modules are compatible and have a positive impact on the experimental results.

#### 5.3.4. Others’ Analyses

**Figure 10:**
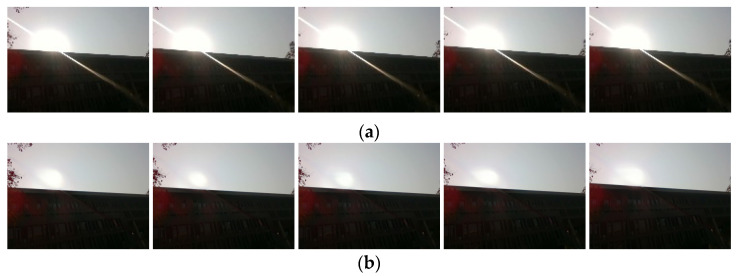
Five consecutive images taken with the D435i camera. (**a**) Image contaminated by the flare; and (**b**) Image after removal using our Sparse Uformer model.

Our approach may be beneficial for tasks that rely on working with visual sensors, and we captured a set of videos using an INTEL REALSENSE D435i camera (Santa Clara, CA, USA). Figure 10a shows five consecutive frames from this set of videos, all of which are disturbed by strong flares, as shown in Figure 2 of Section 1. The ORBSLAM2 system works with a large number of feature points incorrectly extracted to the flares, and Figure 10b shows the flare removal of the above images using our Sparse UFormer model, while we performed flare removal for the two sets of the above images extracted ORB feature points, and Table 5 shows their extraction of erroneous feature points on the flare stripes, respectively, and the results show that the number of erroneous feature points extracted decreases significantly after processing with our model.

## 6. Conclusions

In this paper, we propose a neural network-based method for removing lens flare using a sparse mechanism. Building on the Uformer model, we designed a novel loss function and introduced a sparse transformation block. Our model effectively removes lens flare while preserving the realism of the primary light source, thus enhancing the quality of images for downstream tasks. The proposed method demonstrates state-of-the-art performance through comprehensive qualitative and quantitative results, offering new directions for future research and applications in image processing. Future work will explore the more direct integrations of entropy-based metrics to further enhance the evaluation and optimisation of image processing tasks.

## Figures and Tables

**Table 1:** This table shows the transformation parameters used in our model. The transformations include gamma transformation, rotation (degrees), translation (pixel distance), cutout (degrees), scaling (ratio), blurring (degree), flip, color shift (value), RGB adjustment (ratio), and Gaussian noise (distribution). The ranges for each transformation type are specified.

Transformation Type	Transformation Range
Gamma transformation	[1.8, 2, 2]
Rotation	[0, 2π]
Translation	[−300, 300]
Cutout	[−π/9,π/9]
Scaling	[0.8, 1.5]
Blurring	[0.1, 3]
Flip	Horizontal or vertical
Color shift	[−0.02, 0.02]
RGB adjustment	[0.5, 1.2]
Gaussian noise	$\sigma^{2}\sim 0.01\chi^{2}$

**Table 2:** The evaluation results of our proposed model and other existing state-of-the-art models on the Flare7K++ real test dataset. Bold text in the table indicates the best results. To objectively evaluate the experimental results, our training and testing were entirely based on the publicly available data from Dai [16]. Specifically, the training images included 23,949 background images without flare interference and 7962 flare images, which consisted of 962 real-world flare images and 7000 synthetic flare images. For the test images, both the real test set and the synthetic test set contained 100 images each.

Models	PSNR	SSIM	LPIPS	G-PSNR	S-PSNR
U-Net [24]	27.189	0.894	0.0452	23.527	22.647
HINet [17]	27.548	0.892	0.0464	24.081	22.907
MPRNet [18]	27.036	0.893	0.0481	23.490	22.267
Restormer [19]	27.597	0.897	0.0447	23.828	22.452
UFormer [20]	27.633	0.894	0.0428	23.949	22.603
Uformer + normalised depth [21]	27.662	0.897	0.0422	23.987	22.847
**Sparse-UFormer (ours)**	**27.976**	**0.906**	**0.0413**	**24.243**	**23.529**

**Table 3:** Evaluation results of our proposed model and other existing state-of-the-art models on the Flare7K++ synthetic test dataset. Bold text in the table indicates the best results.

Models	PSNR	SSIM	LPIPS	G-PSNR	S-PSNR
UFormer [20]	29.498	0.962	0.0210	24.686	24.155
Uformer + Normalised Depth [21]	29.573	0.961	0.0205	**24.879**	24.458
**Sparse-UFormer (ours)**	**29.717**	**0.967**	**0.0198**	24.525	**25.014**

**Table 4:** Performance comparison with/without SSIM structural loss and Sparse-transformer. Without loss denotes a model with only Sparse-transformer added to the baseline model. Without Sparse denotes a model with only SSIM structural loss added to the baseline model.

Models	PSNR	SSIM	LPIPS	G-PSNR	S-PSNR
Base	27.633	0.894	0.0428	23.949	22.603
Without loss	27.823	0.895	0.0418	24.082	23.120
Without sparse	27.812	0.902	0.0411	24.201	23.293
Sparse-UFormer (ours)	27.976	0.906	0.0413	24.243	23.529

**Table 5:** Five consecutive frames of flare image erroneous feature point detection result;, the second column in the table is the erroneous feature point extraction results of the flare-contaminated image; the third column is the erroneous feature point extraction results after flare removal using the UFormer baseline algorithm; and the last column is the erroneous feature point extraction results after flare removal using our Sparse Uforeme model.

Pictures	Input	Base	Ours
Picture 1	57	13	2
Picture 2	58	2	2
Picture 3	59	1	4
Picture 4	59	16	9
Picture 5	66	15	3
Avg	60	9	4

## Data Availability

The background images and Flare7K++ datasets are openly available in a public repository. They can be downloaded at https://ceciliavision.github.io/project-pages/reflection.html, accessed on 21 July 2024 and https://github.com/ykdai/Flare7K?tab=readme-ov-file.html, accessed on 8 June 2023.
